# Depression Detection on Reddit With an Emotion-Based Attention Network: Algorithm Development and Validation

**DOI:** 10.2196/28754

**Published:** 2021-07-16

**Authors:** Lu Ren, Hongfei Lin, Bo Xu, Shaowu Zhang, Liang Yang, Shichang Sun

**Affiliations:** 1 Dalian University of Technology Dalian China; 2 State Key Lab for Novel Software Technology Nanjing University Nanjing China; 3 Dalian Minzu University Dalian China

**Keywords:** depression detection, attention network, emotional semantic information, dynamic fusion strategy, natural language processing, social media, emotion, mental health, algorithm, deep learning

## Abstract

**Background:**

As a common mental disease, depression seriously affects people’s physical and mental health. According to the statistics of the World Health Organization, depression is one of the main reasons for suicide and self-harm events in the world. Therefore, strengthening depression detection can effectively reduce the occurrence of suicide or self-harm events so as to save more people and families. With the development of computer technology, some researchers are trying to apply natural language processing techniques to detect people who are depressed automatically. Many existing feature engineering methods for depression detection are based on emotional characteristics, but these methods do not consider high-level emotional semantic information. The current deep learning methods for depression detection cannot accurately extract effective emotional semantic information.

**Objective:**

In this paper, we propose an emotion-based attention network, including a semantic understanding network and an emotion understanding network, which can capture the high-level emotional semantic information effectively to improve the depression detection task.

**Methods:**

The semantic understanding network module is used to capture the contextual semantic information. The emotion understanding network module is used to capture the emotional semantic information. There are two units in the emotion understanding network module, including a positive emotion understanding unit and a negative emotion understanding unit, which are used to capture the positive emotional information and the negative emotional information, respectively. We further proposed a dynamic fusion strategy in the emotion understanding network module to fuse the positive emotional information and the negative emotional information.

**Results:**

We evaluated our method on the Reddit data set. The experimental results showed that the proposed emotion-based attention network model achieved an accuracy, precision, recall, and F-measure of 91.30%, 91.91%, 96.15%, and 93.98%, respectively, which are comparable results compared with state-of-the-art methods.

**Conclusions:**

The experimental results showed that our model is competitive with the state-of-the-art models. The semantic understanding network module, the emotion understanding network module, and the dynamic fusion strategy are effective modules for depression detection. In addition, the experimental results verified that the emotional semantic information was effective in depression detection.

## Introduction

### Background

As defined in the free dictionary, depression refers to the act of depressing or state of being depressed. Depression is usually regarded as one type of mood disorder; the main clinical feature of depression is the significant and persistent mood depression. The depressed patients’ emotion can range from gloomy to grief, low self-esteem, and even to pessimism, which may cause suicidal attempts or behaviors [1]. The World Psychiatric Association set October 10 as the World Mental Health Day in 1992 to strengthen the awareness of the public on mental disorders. The latest report released by the World Health Organization (WHO) pointed out that [2] there were approximately 322 million patients with depression in the world, and the prevalence rate was about 4.4%. The number of patients with depression is growing year by year. From 2005 to 2015, the number of patients with depression worldwide increased by 18.4%. According to the statistics of the WHO [2], depression is one of the 20 main reasons that can cause suicide in the world, accounting for about 1.5% of suicides. It also accounts for the highest proportion of disability among the global diseases and is the main factor of global nonfatal health loss.

With the development of the internet in people’s daily life, people began to share their feelings and problems on social media [3,4] such as Reddit and Twitter. The research of Park et al [5] showed that people with depression tend to post information about depression and even treatment on social media. Thus, we can get a lot of valuable information from social media. If we can judge whether a person has depression based on the information from the internet, it can help the doctors intervene early and avoid the happening of self-injury or suicide. Many researchers, coming from different disciplines such as computer science and psychology, have paid much attention on this topic. In addition, some advanced methods are proposed for depression detection. However, the detection accuracy still needs to be improved.

The goal of depression detection is to classify a person or a post as depressed or not. The performance of depression detection on social media can help with the clinical treatment of depression. This problem needs to be solved. The posts of patients with depression usually contain strong emotions. We give three examples of the textual posts left on Reddit, including two depression-indicative posts and one standard post as follows.

- Example 1: “Today, I feel so horrible, it makes me want to die I made a fool of myself at work, felt so stupid after the meeting so I left work, told the boss I’m sick. Spent the remaining afternoon in bed.” Label: depression
- Example 2: “That feeling when you hate who you are as a person but can’t get yourself to change because you are so used to being like this for the past several years. I’ve become a shitty person. The thought of change seems impossible to me at this point.” Label: depression
- Example 3: “Looking for cool ways to tell parents my wife is pregnant.” Label: nondepression

Examples 1 and 2 contain strong emotional information made by the patients with depression. From example 1, the words, including *horrible*, *die*, and *stupid*, express strong negative emotions of the author. The words *hate* and *shitty* in example 2 also express the author’s strong negative emotions. Example 3 shows the post of a regular user. It does not contain strong negative emotions. As previously mentioned, emotional semantic information usually provides us useful clues for depression detection.

We also counted the proportion of the positive words and the negative words that appeared in the depression-indicative posts and the standard posts of the Reddit data set [6], respectively. The statistical results are shown in Table 1. The percentage of positive emotion words in the table is calculated by 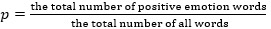. The percentage of negative emotion words was similar. In addition, we calculated the percentages of emotion words in the depression-indicative posts and the standard posts. The depressed users used more negative words than the nondepressed users. At the same time, they used less positive words in their posts than the nondepressed users. It can be concluded from the statistical results that the emotional semantic information may play an effective role for the depression detection task.

**Table 1:** Percentage of emotion words in posts.

Categories	Depression-indicative posts (%)	Standard posts (%)
Positive emotion words	8.62	9.41
Negative emotion words	6.70	4.85

Detecting depression automatically has made some progress. Many existing models detect depression based on the feature engineering such as bag of words [7,8], latent Dirichlet allocation (LDA) [9,10], N-gram [11], Linguistic Inquiry and Word Count (LIWC) dictionary [12], or their combinations [4,13,14]. Bag of words, LDA, and N-gram have been widely used in natural language processing (NLP) for feature extraction and have achieved great progress. LIWC can carry out quantitative analysis on the word categories (especially psychological words) of the text content, including the sentiment, emotion, and so on. Emotion extracted by LIWC is often used in the depression detection task. With the development of deep learning in NLP, more and more studies use deep learning models for depression detection. Orabi et al [15] proposed a method based on deep learning (convolutional neural network [CNN] and recurrent neural network [RNN]) to detect depression. Gui et al [16] proposed a reinforcement learning method based on RNN for depression detection. Although these advanced deep learning based models can extract higher-level semantic information and have achieved great progress, they still lack effective extraction of the emotional semantic information. This may limit the ability of their model because the emotional information may bring effective clues for depression detection, as shown in examples 1 and 2.

Before introducing our model and to understand our paper more conveniently, we give several definitions of concepts, including high-level emotional semantic information, semantic understanding network (SUN), emotion understanding network (EUN), and dynamic fusion strategy.

- High-level emotional semantic information denotes the emotional semantic information that is captured by deep learning.
- SUN is a deep learning method that is used to capture the contextual semantic information in the text for depression detection.
- EUN is a deep learning method that is used to capture the emotional semantic information in the text for depression detection.
- Dynamic fusion strategy denotes a fusion strategy that can fuse positive emotional information and negative emotional information dynamically.

To extract the emotional information effectively, we propose an emotion-based attention network (EAN) for depression detection. Our EAN model mainly contains two modules, including a SUN and an EUN. The SUN module is used to capture the contextual semantic information, which has been widely used in NLP. The EUN module is used to capture the emotional information because the emotional information plays an important role for depression detection as previously mentioned. As shown in Table 1, the depression-indicative posts contained more negative words and less positive words, and the standard posts contained less negative words and more positive words. Thus, we designed the EUN module. The EUN module contains two units, including a positive emotion understanding unit and a negative emotion understanding unit, which are used to extract the positive emotional information and the negative emotional information, respectively. Apart from it, we also propose a dynamic fusion strategy in the EUN module to fuse the positive emotion information and the negative emotion information.

The main contributions of this paper can be summarized as follows:

- We propose a new deep learning framework for depression detection. We also design a special module to explicitly extract the high-level emotion information for depression detection in our framework.
- We take into consideration the positive emotion information and the negative emotion information simultaneously. At the same time, we apply a dynamic fusion strategy to fuse the positive emotion information and the negative information.
- We conduct experiments on the Reddit data set for depression detection. The experiments show our model can get state-of-the-art or comparable performance. The ablation study also verifies the effectiveness of the components proposed in our model.

### Related Work

In this section, we review the related work about depression detection on social media.

In recent years, with the development of social media, more and more people are willing to post their thoughts, emotions, or life details on social media, including Reddit, Twitter, and so on. Park et al [5] showed that people with depression tend to post information about depression and even treatment on social media. Thus, we can get a lot of valuable information from social media. More and more researchers began to analyze the mental health of the users based on the information from social media. As a result, depression detection based on social media has attracted a lot of attention.

De Choudhury et al [17] collected data from Twitter about the users with depression and the regular user, and combined the difference between their behavior on social media (depressed users manifested as decreased social activities, increased negative emotions and self-concern, a high degree and increased expression of religious thoughts, etc) and established a characteristic model for depression detection. Park et al [18] tested for users with depression through social media and conducted semistructured face-to-face interviews with 14 active users. The study concluded that users with depression regarded social media as a platform for social awareness and emotional sharing, while users with nondepression regarded social media as a platform for sharing information. Thus, emotional information is important in the task of detecting depression in social media.

Most of the existing methods for depression detection are based on feature engineering. LIWC is usually used to extract individual psychological states, such as positive and negative emotions, pronouns, and so on. Therefore, LIWC was often used for the depression detection task [4,12-14]. Kang et al [19] proposed a multimodal method for depression detection including text analysis, a word-based emoticon analysis, and a support vector machine–based image classifier. The authors applied visual sentiment ontology [20] and SentiStrength dictionaries to build a mood lexicon for emoticon analysis to enhance the results of depression detection. Shen et al [21] extracted six depression-related feature groups (including social network feature, user profile feature, visual feature, emotional feature, topic-level feature, and domain-specific feature) for depression detection. Hiraga [22] extracted linguistic features for depression detection, including character n-grams, token n-grams, and lemmas and selected lemmas. Hussain et al [3] developed an application called the Socially Mediated Patient Portal. The application could generate a series of features for depression detection.

Shneidman [23] presented depression that tended to be closely related to suicide. De Choudhury et al [24] analyzed Reddit users’ posts on the topic of mental health that later turned to the topic of suicidal thoughts. This turn could be predicted by traits such as self-focus, poor language style, reduced social engagement, and expressions of despair or anxiety. Yates et al [25] proposed a neural framework for depression detection, and they presented that self-harm was closely related to depression. The Conference and Labs of Evaluation Forum for Early Risk Prediction (CLEF eRISK) is a public competition about different areas such as health and safety [26]. CLEF eRISK 2018 is about the early detection of depression and anorexia [8,27]. CLEF eRISK 2019 is about the severity of symptoms of depression, self-injury, and anorexia [28].

Different from traditional feature engineering-based methods, deep learning methods mostly apply end-to-end models. Yates et al [25] proposed a neural framework based on a CNN for depression detection. Orabi et al [15] proposed a neural method based on a CNN and RNN for depression detection. Song et al [29] proposed a neural network that was named the feature attention network for depression detection. Gui et al [16] proposed a reinforcement learning method based on long short-term memory (LSTM) for depression detection. Ray et al [30] proposed a multilevel attention network to fuse the features from the multimodal for depression detection.

According to previous research on depression detection, it can be concluded that the emotional information is important in the task of depression detection. In addition, deep learning can take high-level semantic information into account, but the current deep learning methods for depression detection still lack effective extraction of the emotional semantic information. Thus, we propose a deep learning model to consider the high-level emotional information that is captured by the deep learning method for depression detection, which is named the EAN.

The structure of this paper is organized as follows. The Introduction section introduced the background and related work. The Methods section shows the details of the proposed model. The Results section gives the experiments in this paper. The Discussion section shows the conclusions and future work.

## Methods

### Data Sets

As a newly developed social media, Reddit has become a widely popular web-based discussion forum. Reddit users can discuss a variety of topics on this web-based platform anonymously. The topics discussed on the platform can be arranged in more than a million discussion groups. Due to the large amount of discussion text, Reddit attracts many researchers to conduct their studies with the data on the Reddit platform. Pirina and Çöltekin [6] built a data set for depression detection based on Reddit, which was named the Reddit data set. The samples in the Reddit data set [6] are collected from the Reddit platform. The Reddit data set [6] contains 1293 depression-indicative posts and 549 standard posts.

**Figure 1:**
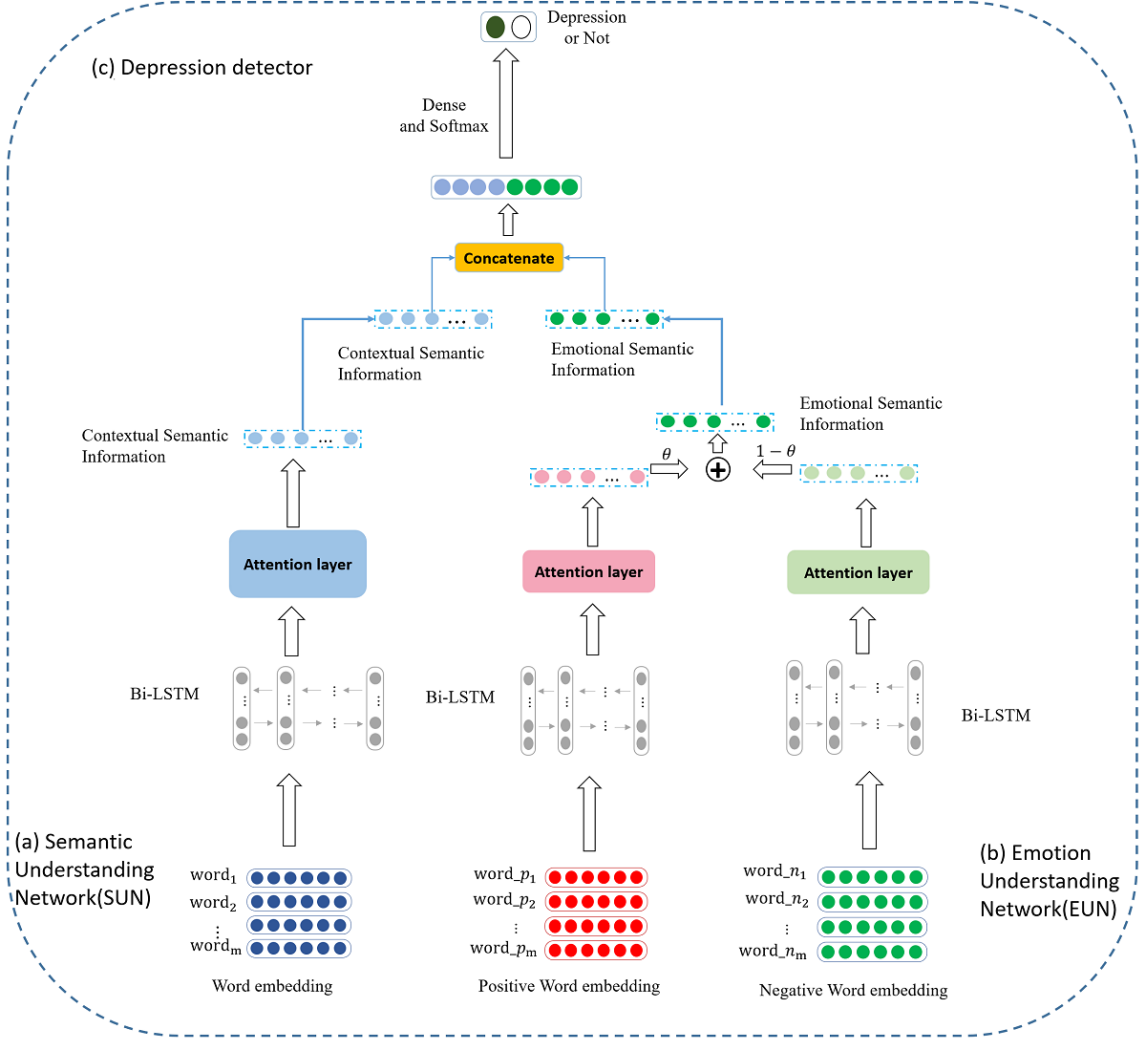
The architecture of the emotion-based attention network model. There are two parts in our model, including a SUN and an EUN. bi-LSTM: bidirectional long short-term memory.

We preprocessed the Reddit data set, such as removing the stop words. We then counted the occurrence number of each word for the depression-indicative posts and the standard posts. We sorted the words according to the statistics and show the top of the word lists in Figure 1. We also counted the occurrence number of the positive emotion words and the negative emotion words for the depression-indicative posts and the standard posts. For all of the words, the positive emotion words and the negative emotion words with high frequency of occurrence are also shown in Textbox 1.

As shown in Textbox 1, from the most commonly used words of the depressed users, we can see many negatives words are also included in the most commonly used words such as depression or fucking. The most common words for nondepressed people are commonly used words in daily life. As can be seen from the list of negative words with high frequency of occurrence used by users with depression, the negative words used by users with depression are more intense than the negative words appearing in the posts of nondepressed users, such as suicide, die, kill, and hate.

**Data analysis.**

**Depression-indicative posts**

- All text: i’m, like, feel, want, get, know, even, really, people, life, i’ve, one, time, think, would, never, depression, me, can’t, go, going, things, don’t, much, friends, make, good, it, still, could, back, anyone, years, anything, always, every, got, someone, fucking, help, day, see, something, work, ever, need, feeling, everything, talk, year
- Positive: friends, good, work, help, better, happy, job, love, hard, friend, family, care, wanted, best, sleep, sure, self, mind, understand, new, mental, hope, social, money, high, remember, working, reason, okay, close, real, together, great, normal, deal, believe, change, enjoy, birthday, honestly, nice, motivation, advice, loved, therapist, happiness, fun, boyfriend, saying, big
- Negative: depression, depressed, bad, fucking, nothing, alone, hate, shit, stop, lost, worse, anxiety, fuck, tired, sad, die, suicide, kill, relationship, wrong, pain, suicidal, problems, old, sorry, cry, lonely, therapy, hurt, stupid, constantly, issues, sick, crying, problem, afraid, weird, reddit, hospital, worst, hang, illness, dead, scared, dark, broken, shitty, broke, miserable, died

**Standard posts**

- All text: like, i’m, know, friend, would, feel, really, friends, want, time, get, one, even, said, always, never, told, got, family, go, things, me, think, best, make, mom, going, people, years, talk, also, still, back, something, much, see, say, could, i’ve, dad, tell, since, don’t, started, us, me, it, made, help, parents
- Positive: friend, friends, family, best, sister, help, friendship, work, brother, good, new, sure, love, wanted, saying, together, advice, father, close, money, boyfriend, kids, care, hard, better, mad, understand, job, basically, happy, great, deal, child, high, moved, believe, fun, social, mind, baby, conversation, eventually, reason, married, big, change, spend, real, normal, nice
- Negative: bad, wrong, nothing, old, hang, problem, stop, hurt, upset, sorry, shit, issues, lost, alone, cut, angry, hate, problems, worse, depression, weird, sick, constantly, anxiety, sad, tired, annoyed, broke, bitch, scared, died, hell, afraid, crying, cancer, toxic, ignore, pregnant, lose, difficult, wait, fault, depressed, horrible, awkward, selfish, reply, fuck, confused, reddit

### Overview of the EAN Model

In this section, we introduce the proposed model for depression detection briefly, which is called the EAN, as shown in Figure 1. The proposed EAN model mainly contains two parts, including a SUN and an EUN. The SUN module is used to capture the contextual semantic information in the depression-indicative posts. The EUN module is used to capture the emotional semantic information in the depression-indicative posts. Finally, we concatenated the features captured by the two parts and judged whether the text is depression-indicative or not by the depression detector. We give details on the SUN, the EUN, and the loss function next.

### Semantic Understanding Network

The SUN was used to capture the contextual semantic information in the text for depression detection. There are three layers in the SUN module, including the word encoding layer, context encoding layer, and attention mechanism (Att) layer. We will introduce these three layers in more details.

#### Word Encoding Layer

We will introduce the word encoding layer in the SUN module briefly. The input of our task is text. The text can be denoted as w = {*w*_1_, *w*_2_, ..., *w_n_*}, where n denotes the length of the text, and *w_i_* denotes the word in the text. In NLP tasks, words are usually mapped to the form of word vectors. Inspired by it, we also encoded every word into d-dimension word vector. We applied the pretrained Global Vectors for Word Representation (GloVe) [31] here. We then can get the textual representation S = *R^n^* ^×^ *^d^*, where n is the textual length and d is the dimension of the word.

#### Context Encoding Layer

The context encoding layer was used to obtain contextual information. Bidirectional long short-term memory (Bi-LSTM) [32] was widely used in NLP tasks to capture the contextual information. Inspired by this, we applied Bi-LSTM in the context encoding layer. Bi-LSTM contains a forward directional LSTM and a backward directional LSTM. The output 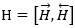 Bi-LSTM contains two parts, including the forward LSTM output 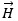 and the backward LSTM output 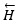.

LSTM was proposed by Hochreiter and Schmidhuber [33] and was used to capture the forward information in the text. LSTM cannot capture the backward information; therefore, Bi-LSTM was proposed. LSTM owns three gates and one cell, including an input gate *i_t_*, a forget gate *f_t_*, an output gate *o_t_*, and a memory cell *c_t_*. The operations of LSTM are as following.


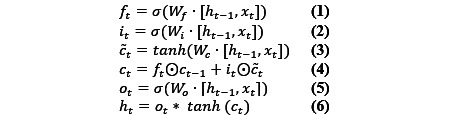


Where *x_t_* is the current input word vector, 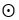 means the elementwise multiplication operation, and *σ* means the sigmoid function. *W_f_*, *W_i_*, *W_c_*, and *W_o_* represent the parameters that can be trained in the training processing. *h_t_* is the hidden state vector. 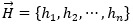 is the output of LSTM. More details on LSTM can be found in Hochreiter and Schmidhuber [33], and the output of Bi-LSTM is H = [*H*_1_, *H*2, ..., H*_n_*].

#### Attention Mechanism Layer

The input of the Att layer is H = [*H*_1_, *H*_2_, ..., *H_n_*]. The Att is used to assign higher weights on the important words. We applied the Att to capture the important words in the depression-indicative posts for the depression detection task. The operations of the Att are based on the following equations:


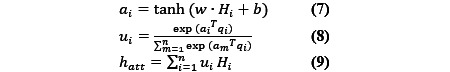


Where *H_i_* is the hidden state vector of Bi-LSTM, *w* and *q_i_* are the weighted matrices, and *h_att_* is the output of the Att.

### Emotion Understanding Network

Many research papers [19-21] and their experiments have proven the effectiveness of emotional feature in depression detection tasks. Inspired by this, we considered the high-level emotional semantic information in the depression-indicative posts based on the EUN. The EUN was used to capture the emotional semantic information in the text for depression detection. There are three layers in the EUN module, including the input layer, emotion encoding layer, and emotion fusion layer. We introduce these three layers in more detail in the following sections.

#### Input Layer

In this section, we introduce the inputs of the EUN module. The inputs include a positive emotion part and a negative emotion part. We applied the SenticNet application programming interface to divide the original texts into a positive emotional part and a negative emotional part. These two emotional parts are also mapped into a matrix of word vectors as in the word encoding layer in the SUN module, named *R_pos_* and *R_neg_*, respectively.

#### Emotion Encoding Layer

The emotion encoding layer is to encode the positive emotional information and the negative emotional information. *R_pos_* and *R_neg_* act as the inputs of the emotion encoding layer. There are two units in the emotion encoding layer, including the positive emotion understanding unit and the negative emotion understanding unit. These two units are used to capture positive emotional information and negative emotional information, respectively. We also applied Bi-LSTM to capture the contextual emotional information and the Att to capture the important emotions in the text in both units. The operations of Bi-LSTM and the Att are the same as the EUN module. We can get *h_pos_* from the positive emotion understanding unit and *h_neg_* from the negative emotion understanding unit.

#### Emotion Fusion Layer

The goal of the emotion fusion layer is to fuse the positive emotional information and the negative emotional information for depression detection. We get the positive emotional information *h_pos_* and the negative emotional information *h_neg_* from the emotion encoding layer, which can be learned in the training processing. Considering the difference of each text, we designed a dynamic fusion strategy that can dynamically fuse the positive emotional information *h_pos_* and the negative emotional information *h_neg_*. Inspired by the Att, we design a random floating point number θ∈[0,1]. It can be trained during the training. We can get the output *h_emo_* of the EUN module with the following formula:

*h_emo_* = θ * *h_pos_* + (1 – θ) * *h_neg_***(10)**

### Loss Function

As previously described, we get the contextual semantic information *h_att_* from the SUN module and the emotional semantic information *h_emo_* from the EUN module. In this section, we applied a concatenation operation to fuse the contextual semantic information *h_att_* and the emotional semantic information *h_emo_* as the final representation *f_final_*:

*f_final_* = concatenate[*h_att_*; *h_emo_*] **(11)**

Accordingly, the final classification decision for depression detection is formulated by the softmax function:

y = softmax(W ∙ *f_final_* + b) **(12)**

The cross-entropy loss was used for depression detection in our model. The training goal was to minimize the loss.

## Results

### Implementation Details and Metrics

The unit size of Bi-LSTM in our experiments was 64. We applied the pretrained 300-dimension word embedding (GloVe) in the word encoding layer. In addition, the optimization function was Adam, and the batch size was 128. Following Tadesse et al [4], we also applied a 10-fold cross validation in our experiments; 90% of posts in the data sets were used as our training set, and the other 10% of posts were used as the testing set.

We applied the standard metrics, including accuracy, precision, recall, and F1-score, to evaluate the effectiveness of our model for depression detection. F1 is defined as follows:


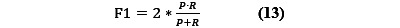


### Comparison With Existing Methods

We compared the results of our model with many state-of-the-art methods on the Reddit data set. We compared it with the baselines, including LIWC, LDA, unigram, bigram, LIWC + LDA + unigram, LIWC + LDA + bigram [4], LSTM, Bi-LSTM, and Bi-LSTM + Att.

- LIWC: Tadesse et al [4] extracted the linguistic features and the psychological features based on LIWC [34] for depression detection.
- LDA: Tadesse et al [4] extracted 70 dimensional characteristics of the topic based on LDA. It can be helpful in discovering its underlying topic structures for depression detection.
- Unigram: Tadesse et al [4] extracted 3000 dimensional characteristics based on unigram in term frequency–inverse document frequency (TF–IDF) for depression detection.
- Bigram: Tadesse et al [4] extracted 2736 dimensional characteristics based on bigram in TF–IDF for depression detection.
- LIWC + LDA + unigram: The model is based on the aforementioned characteristics, including LIWC, LDA, and unigram, for depression detection.
- LIWC + LDA + bigram: The model is based on the aforementioned characteristics, including LIWC, LDA, and bigram, for depression detection.
- LSTM: LSTM was proposed by Hochreiter and Schmidhuber [33]. We applied the same word embedding in this paper, and the unit size was 128.
- Bi-LSTM: The Bi-LSTM was proposed by Graves et al [32]. We applied the same setting and the same word embedding in this paper.
- Bi-LSTM + Att: The model is based on Bi-LSTM and the Att.
- EAN: This model is proposed in this paper, which considers emotional semantic information based on deep learning.

As shown in Table 2, the results based on deep learning are generally higher than the results based on feature engineering methods. It is because deep learning can capture the higher semantic information of texts. In addition, we can also get the following conclusions.

The results based on bigram (bigram and LIWC + LDA + bigram) were higher than unigram (unigram and LIWC + LDA + unigram). It can be concluded that contextual information can improve the results of the model. The results based on Bi-LSTM were higher than LSTM. it can be concluded that considering bidirectional contextual semantic information is necessary. The results based on Bi-LSTM + Att were higher than Bi-LSTM; it can be proven that the Att is effective for the depression detection task. The proposed EAN model got the higher results because we took into consideration both the contextual semantic information and the emotional semantic information.

**Table 2:** Results compared with the existing models.

Model	Accuracy (%)	Precision (%)	Recall (%)	F1 (%)
LIWC^a,b^	70	74	71	72
LDA^b,c^	75	75	72	74
Unigram^b^	70	71	95	81
Bigram^b^	79	80	76	78
LIWC + LDA + unigram^b^	78	84	79	81
LIWC + LDA + bigram^b^	91	90	92	91
LSTM^d^	87.03	90.30	91.67	90.98
Bi-LSTM^e^	86.46	88.08	95	91.41
Bi-LSTM + Att^f^	88.59	90.41	94.96	92.63
EAN^g^ (our model)	91.3	91.91	96.15	93.98

^a^LIWC: Linguistic Inquiry and Word Count.

^b^Indicates that the results are shown in the literature [4].

^c^LDA: latent Dirichlet allocation.

^d^LSTM: long short-term memory.

^e^Bi-LSTM: bidirectional long short-term memory.

^f^Att: attention mechanism.

^g^EAN: emotion-based attention network.

### Detail Analysis

In this section, we analyze the effectiveness of the two modules (SUN and EUN), the effectiveness of different emotional semantic information, and the effectiveness of the dynamic fusion strategy.

#### The Effectiveness of SUN and EUN

**Figure 2:**
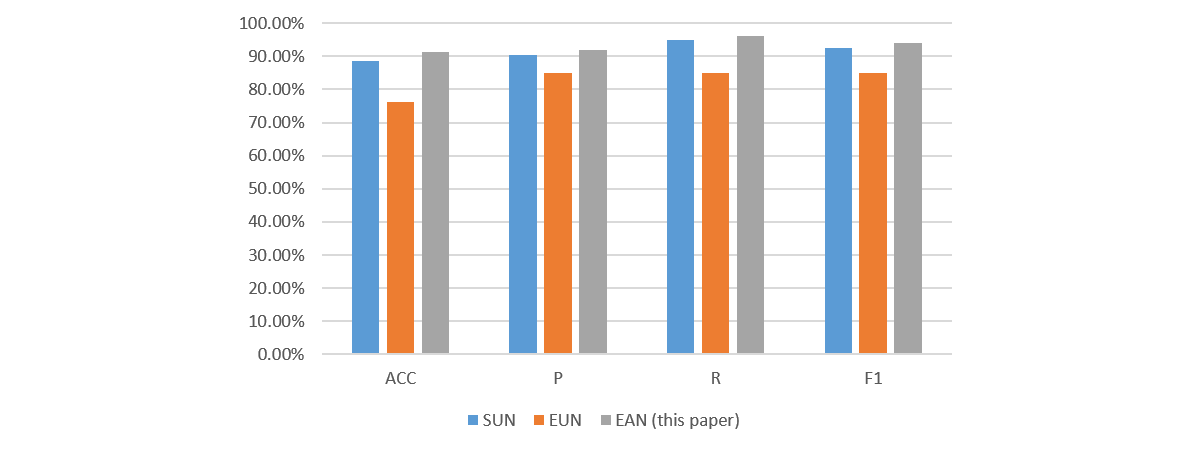
The effectiveness of the SUN and EUN. EAN: emotion-based attention network; EUN: emotion understanding network; SUN: semantic understanding network.

To verify the effectiveness of SUN and EUN, we designed a series of experiments. SUN means the proposed EAN model without the EUN module. EUN means the proposed EAN model without the SUN module. As shown in Figure 2, the EUN module obtained the worst results. This is because the model only considers the emotional semantic information without the complete semantic information. It verifies the effectiveness of our SUN module. The results of the EAN model were higher than the SUN module, which further verifies the effectiveness of our EUN module.

#### The Effectiveness of Different Emotional Semantic Information

**Figure 3:**
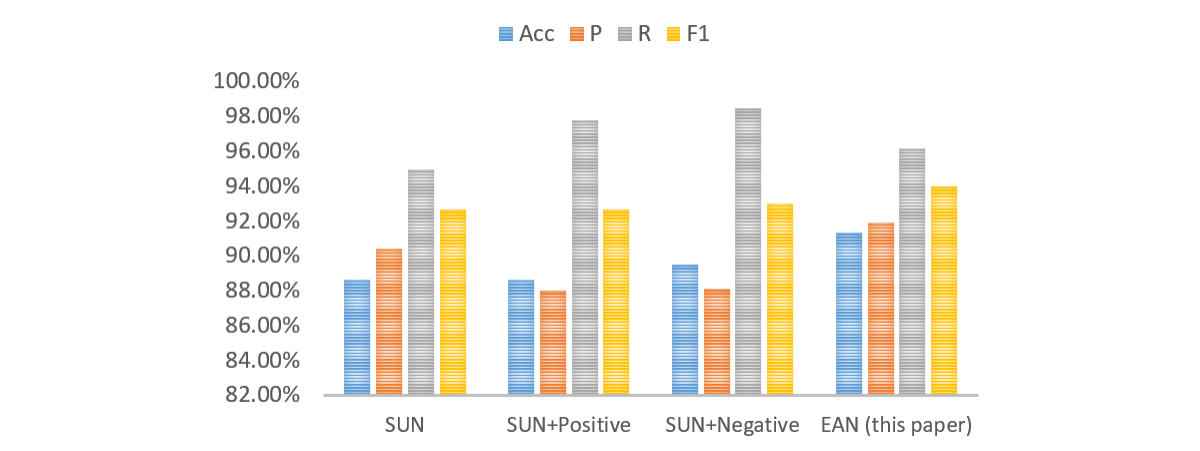
The effectiveness of different emotional semantic information. Acc: accuracy; EAN: emotion-based attention network; P: precision; R: recall; SUN: semantic understanding network.

To verify the effectiveness of different emotional semantic information, we designed a series of experiments, including without emotion (SUN), without positive emotion (SUN + negative), and without negative emotion (SUN + positive). As shown in Figure 3, the results of the SUN + positive model and the SUN module were similar. It indicates that positive emotions have less effect on the model. Although the EAN model does not obtain the best recall value, it obtained the best *P* value, ACC value, and F1 value. From the experiments, our proposed EAN model obtained the best result compared to the three aforementioned baseline models. It also verified the effectiveness of each proposed module in our framework.

#### The Effectiveness of the Dynamic Fusion Strategy

**Figure 4:**
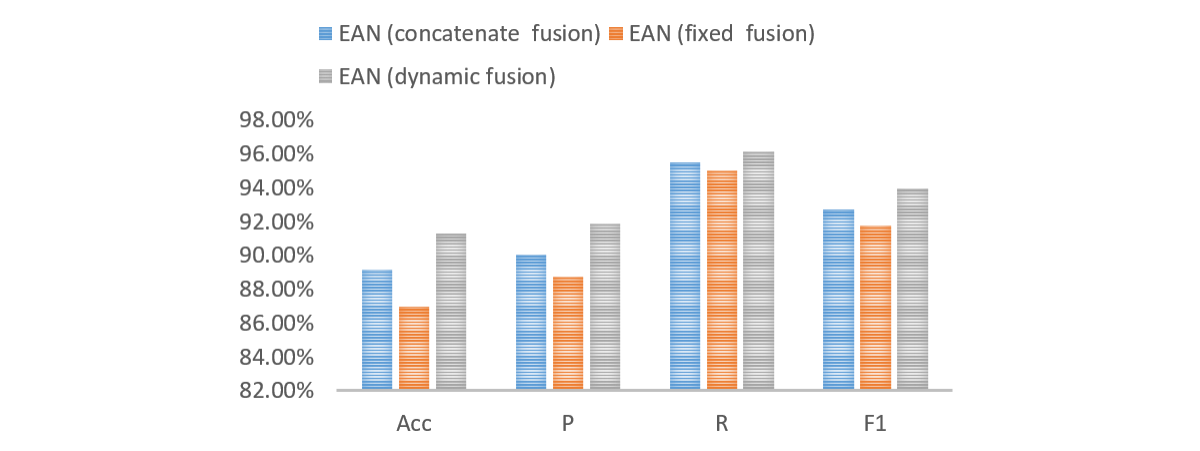
The effectiveness of the dynamic fusion strategy. Acc: accuracy; EAN: emotion-based attention network; P: precision; R: recall.

To verify the effectiveness of the dynamic fusion strategy, we designed a series of experiments including the EAN model with the concatenate fusion strategy, the EAN model with the fixed fusion strategy, and the EAN model with the dynamic fusion strategy. The EAN (concatenate fusion) model applies the concatenate operation in the emotion fusion strategy. The EAN (fixed fusion) model applies the fixed fusion operation in the emotion fusion layer. The θ in equation 10 is fixed at 0.5. The EAN (dynamic fusion) model is the model proposed in this paper. As shown in Figure 4, the dynamic fusion method had the best results.

In this section, we designed a series of experiments to verify the effectiveness of the proposed EAN model, including the two modules in the EAN model, the different emotional semantic information, and the dynamic fusion method.

**Figure 5:**
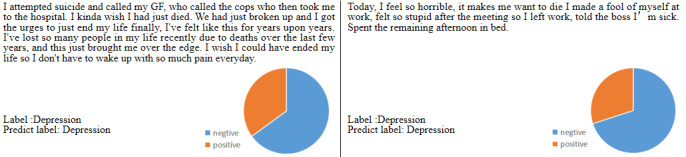
The visualization of the θ in the dynamic fusion strategy. GF: girlfriend.

Some visualization results of the θ to illustrate the effectiveness of the proposed dynamic fusion strategy intuitively are shown in Figure 5. As shown in Figure 5, the examples are both depression-indicative posts. The pie chart indicates the value of the θ in the dynamic fusion strategy. We can see from the results that in the depression-indicative posts, the negative emotional information can be paid more attention.

## Discussion

### Conclusion

Depression attracts more and more attention from people and organizations now. With the development of computer technology, some researchers are trying to use computers to automatically identify people who are depressed. In this paper, we proposed an EAN model to explicitly extract the high-level emotion information for the depression detection task. The proposed EAN model consists of the SUN and the EUN. In the proposed model, we took into consideration the positive emotion information and the negative emotion information simultaneously. At the same time, we applied a dynamic fusion strategy to fuse the positive emotion information and the negative information. The experimental results verified that the emotional semantic information is effective in depression detection.

### Future Work

According to WHO statistics, depression is one of the main causes of suicide in the world. We will focus on the relationship between depression and suicide. We will try to combine suicide detection with depression detection in our future work to improve the performance of both tasks by multitask learning. In addition, the future work will be combined with self-reported depressive symptoms or clinical diagnosis. Hopefully, our study can provide some technical supports in the field of health care.

## Abbreviations

Att: attention mechanism
Bi-LSTM: bidirectional long short-term memory
CLEF eRISK: Conference and Labs of Evaluation Forum for Early Risk Prediction
CNN: convolutional neural network
EAN: emotion-based attention network
EUN: emotion understanding network
GloVe: Global Vectors for Word Representation
LDA: latent Dirichlet allocation
LIWC: Linguistic Inquiry and Word Count
LSTM: long short-term memory
NLP: natural language processing
RNN: recurrent neural network
SUN: semantic understanding network
TF–IDF: term frequency–inverse document frequency
WHO: World Health Organization

## References

[1] Friedrich M (2017). Depression is the leading cause of disability around the world. JAMA.

[2] (2017). Depression and other common mental disorders: global health estimates. World Health Organization.

[3] Hussain J, Satti FA, Afzal M, Khan WA, Bilal HSM, Ansaar MZ, Ahmad HF, Hur T, Bang J, Kim J, Park GH, Seung H, Lee S (2019). Exploring the dominant features of social media for depression detection. J Inf Sci.

[4] Tadesse MM, Lin H, Xu B, Yang L (2019). Detection of depression-related posts in Reddit social media forum. IEEE Access.

[5] Park M, Cha C, Cha M (2012). Depressive moods of users portrayed in twitter.

[6] Pirina I, Çöltekin Ç (2018). Identifying depression on reddit: The effect of training data.

[7] Nadeem M Identifying depression on twitter. arXiv..

[8] Paul S, Kalyani JS, Basu T (2018). Early detection of signs of anorexia and depression over social media using effective machine learning frameworks.

[9] Maupomé D, Meurs MJ (2018). Using topic extraction on social media content for the early detection of depression.

[10] Resnik P, Armstrong W, Claudino L, Nguyen T, Nguyen VA, Boyd-Graber J (2015). Beyond ldaxploring supervised topic modeling for depression-related language in Twitter.

[11] Benton A, Mitchell M, Hovy D Multi-task learning for mental health using social media text. arXiv..

[12] Coppersmith G, Dredze M, Harman C, Hollingshead K (2015). From ADHD to SAD: analyzing the language of mental health on twitter through self-reported diagnoses.

[13] Wolohan JT, Hiraga M, Mukherjee A, Sayyed ZA (2018). Detecting linguistic traces of depression in topic restricted text: attending to self-stigmatized depression with NLP.

[14] Tyshchenko Y (2018). Depression and anxiety detection from blog posts data. CORE.

[15] Orabi AH, Buddhitha P, Orabi MH, Inkpen D (2018). Deep learning for depression detection of Twitter users.

[16] Gui T, Zhang Q, Zhu L, Zhou X, Peng M, Huang X, Sun M, Huang X, Ji H, Liu Z, Liu Y (2019). Depression detection on social media with reinforcement learning. Chinese Computational Linguistics 18th China National Conference, CCL 2019, Kunming, China, October 18–20, 2019, Proceedings.

[17] De Choudhury M, Gamon M, Counts S, Horvitz E (2013). Predicting depression via social media.

[18] Park M, McDonald D, Cha M (2013). Perception differences between the depressed and non-depressed users in Twitter.

[19] Kang K, Yoon C, Kim EY (2016). Identifying depressive users in twitter using multimodal analysis.

[20] Borth D, Ji R, Chen T, Breuel T, Chang SF (2013). Large-scale visual sentiment ontology and detectors using adjective noun pairs. Proceedings of the 21st ACM International Conference on Multimedia.

[21] Shen G, Jia J, Nie L, Feng F, Zhang C, Hu T, Chua TS, Zhu W (2017). Depression detection via harvesting social media: a multimodal dictionary learning solution. Proceedings of the Twenty-Sixth International Joint Conference on Artificial Intelligence.

[22] Hiraga M (2017). Predicting depression for Japanese blog text.

[23] Shneidman ES (1993). Suicide as psychache. J Nerv Ment Dis.

[24] De Choudhury M, Kiciman E, Dredze M, Coppersmith G, Kumar M (2016). Discovering shifts to suicidal ideation from mental health content in social media. Proceedings of the 2016 CHI Conference on Human Factors in Computing Systems.

[25] Yates A, Cohan A, Goharian N Depression and self-harm risk assessment in online forums. arXiv..

[26] Losada DE, Crestani F, Parapar J, Jones GJF, Lawless S, Gonzalo J, Kelly L, Goeuriot L, Mandl T, Cappellato L, Ferro N (2017). eRISK 2017: CLEF lab on early risk prediction on the internet: experimental foundations. Experimental IR Meets Multilinguality, Multimodality, and Interaction 8th International Conference of the CLEF Association, CLEF 2017, Dublin, Ireland, September 11–14, 2017, Proceedings.

[27] Trotzek M, Koitka S, Friedrich CM (2020). Utilizing neural networks and linguistic metadata for early detection of depression indications in text sequences. IEEE Trans Knowledge Data Eng.

[28] Losada DE, Crestani F, Parapar J, Crestani F, Braschler M, Savoy J, Rauber A, Müller H, Losada DE, Bürki GH, Cappellato L, Ferro N (2019). Overview of eRisk 2019 early risk prediction on the internet. Experimental IR Meets Multilinguality, Multimodality, and Interaction: 10th International Conference of the CLEF Association, CLEF 2019, Lugano, Switzerland, September 9–12, 2019, Proceedings.

[29] Song H, You J, Chung JW, Park JC (2018). Feature attention network: interpretable depression detection from social media.

[30] Ray A, Kumar S, Reddy R, Mukherjee P, Garg R (2019). Multi-level attention network using text, audio and video for depression prediction. Proceedings of the 9th International on Audio/Visual Emotion Challenge and Workshop.

[31] Pennington J, Socher R, Manning C (2014). GloVe: global vectors for word representation. Proceedings of the 2014 Conference on Empirical Methods in Natural Language Processing.

[32] Graves A, Jaitly N, Mohamed AR (2013). Hybrid speech recognition with Deep Bidirectional LSTM.

[33] Hochreiter S, Schmidhuber J (1997). Long short-term memory. Neural Comput.

[34] Pennebaker JW, Booth RJ, Boyd RL, Francis ME (2001). Linguistic Inquiry and Word Count: LIWC2015. Pennebaker Conglomerates.

